# Assessing the Potential of Land Use Modification to Mitigate Ambient NO_2_ and Its Consequences for Respiratory Health

**DOI:** 10.3390/ijerph14070750

**Published:** 2017-07-10

**Authors:** Meenakshi Rao, Linda A. George, Vivek Shandas, Todd N. Rosenstiel

**Affiliations:** 1 School of the Environment, Portland State University, Portland, OR 97207, USA; mrao@pdx.edu; 2 Nohad A. Toulan School of Urban Studies and Planning, Portland State University, Portland, OR 97207, USA; vshandas@pdx.edu; 3 Department of Biology, Portland State University, Portland, OR 97207, USA; rosensti@pdx.edu

**Keywords:** nitrogen dioxide, air pollution, land use regression, random forest, health, BenMAP

## Abstract

Understanding how local land use and land cover (LULC) shapes intra-urban concentrations of atmospheric pollutants—and thus human health—is a key component in designing healthier cities. Here, NO_2_ is modeled based on spatially dense summer and winter NO_2_ observations in Portland-Hillsboro-Vancouver (USA), and the spatial variation of NO_2_ with LULC investigated using random forest, an ensemble data learning technique. The NO_2_ random forest model, together with BenMAP, is further used to develop a better understanding of the relationship among LULC, ambient NO_2_ and respiratory health. The impact of land use modifications on ambient NO_2_, and consequently on respiratory health, is also investigated using a sensitivity analysis. We find that NO_2_ associated with roadways and tree-canopied areas may be affecting annual incidence rates of asthma exacerbation in 4–12 year olds by +3000 per 100,000 and −1400 per 100,000, respectively. Our model shows that increasing local tree canopy by 5% may reduce local incidences rates of asthma exacerbation by 6%, indicating that targeted local tree-planting efforts may have a substantial impact on reducing city-wide incidence of respiratory distress. Our findings demonstrate the utility of random forest modeling in evaluating LULC modifications for enhanced respiratory health.

## 1. Introduction

Cities have increasingly become the nexus of air pollution due to emissions from anthropogenic activities within the cities [1]. The dispersion of these emissions is not uniform across the urban landscape leading to high spatial variation in ambient air pollution concentrations [2,3]. Local concentrations of air pollutants are affected not just by the strength of local emissions and formation of secondary air pollutant through atmospheric chemistry, but also by land use and land cover (LULC) features which influence air flow and hence transport of these pollutants. Urban zoning and other siting policies affect the proximity of a location to emission sources, while the quantity of emissions associated with each LULC category is controlled through permitting, policies, and urban planning. Atmospheric scientists capture the spatial variation of atmospheric pollutants within a city or region using a wide variety of techniques [2,4] such as land use regression (LUR) [5,6,7]; geo-statistical techniques including kriging [8,9,10]; modeling simulations such as dispersion models [11,12,13] and atmospheric chemistry & transport models (ACTMs) [14,15]; computational fluid dynamics models (CFDs) [16]; and other emerging techniques [17,18,19,20]. All these modeling techniques explicitly include LULC and terrain data as model inputs, acknowledging the influence of LULC on local ambient concentrations of air pollutants. In our rapidly urbanizing world, where ambient air pollution is recognized as a leading environmental health risk [1], the role of land use in modulating ambient concentrations of air pollution gives rise to a timely and relevant question: to what extent can urban land use be managed to decrease local air pollution, and consequently, its impact on human health? 

Globally, managing land use to reduce greenhouse gas emissions or air pollution is not a new concept. Hong Kong requires an air ventilation assessment for all publicly funded construction to mitigate the stagnant wind conditions that could allow accumulation of air pollutants or air-borne diseases like SARS [21] in its urban canyons. In California, schools are required to be located more than a quarter mile away from sources of potentially hazardous air pollutants [22]. Many cities actively seek to reduce vehicle miles traveled (VMT) to reduce CO_2_ emissions and manage regional air quality [23,24,25]. However, even though LULC has been regulated to reduce or avoid exposure to emissions, the role of local LULC modifications on ambient concentrations through dispersion or deposition of air pollutants, has—with few exceptions [26,27,28,29,30]—not been systematically investigated.

Assessing the impact of local land use on air pollution presents some unique challenges. Simulation models, although theoretically ideal for studying the effect of LULC on ambient air pollutions, suffer from a lack of validated emission inventories at a fine spatial scale (~1 km) [31,32]. Additionally, ACTMs require a very high level of expertise and computational power to be run at this fine intra-urban resolution. Geo-statistical models such as LUR, on the other hand, have been successfully used to capture the fine spatial scale variation of urban air pollutants in cities across the world [33,34,35,36,37]. However, though these statistical regression techniques are excellent at developing predictive models, the correlated nature of most land use variables makes it difficult to use these same techniques to isolate the impact of individual LULC categories on local ambient air pollution concentrations [38,39]. Nevertheless, with rising urbanization and the growing recognition of the negative economic and health impacts of air pollution [40,41], there exists an urgent demand for developing techniques that can help us evaluate the role of LULC and LULC modifications on urban air pollutants, especially techniques that do not require a high level of expertise or computational power, and thus are readily accessible to all stakeholders engaged in managing or minimizing local concentrations of ambient air pollutants [42,43]. 

In this paper we use a technique called random forest that can be used to investigate the association of air pollution with individual LULC categories. Random forest [44,45] is a powerful ensemble-based data mining technique that makes minimal assumptions about the independence or underlying distribution of the predictor variables. It has been widely adopted and successfully applied in many domains, including bioinformatics and medical research [46,47,48,49], land use classification [50,51] and ecological modeling [52,53], and is being increasingly applied in the field of for air pollution as well [19,20,54]. The strengths of random forest lie in that fact that it makes minimal assumptions about the underlying data distribution or correlation of predictors, has the ability to deal with noisy and missing data, and can deal with the “large number of predictors, small number of observations” situation typical of urban air quality and other environmental studies in general. Further, as new environmental sensor technologies are more widely deployed [55,56] , providing streams of intra-urban observational data, the need for new methodologies to analyze the flood of incoming data and make it accessible and informative to scientists, planners and the public alike, will be intensified.

We exploit the ability of random forest to handle a large number of correlated predictors in order to examine the response of the US criteria pollutant nitrogen dioxide (NO_2_) [57] to LULC and LULC modifications in the Portland-Hillsboro-Vancouver metropolitan region. We study NO_2_ as it is one of the more easily measured of the US criteria pollutants. It is also a strong marker of anthropogenic combustion-related pollution, and a precursor to two other criteria pollutants, ozone and fine particulate matter [58,59]. In this study, we first use random forest to develop a model of NO_2_, fitting NO_2_ observations with LULC variables. Since random forest is not yet widely used to model high spatial resolution urban air pollution, we also assess the performance of the land use random forest (LURF) model. Leveraging the ability of random forest to handle correlated predictors, we use the LURF model to investigate the association of ambient NO_2_ concentrations with individual LULC categories. Further, by using the LURF NO_2_ models in conjunction with BenMAP [60,61,62], a health impact assessment tool from the US Environmental Protection Agency (EPA), we estimate the relative health impacts associated with each LULC category through its association with NO_2_. Finally, we investigate the potential of LULC modifications as a strategy for mitigating the health impacts of NO_2_ by undertaking a sensitivity analysis, examining the change in NO_2_ associated with the systematic variation of selected LULC categories, and using BenMAP to estimate the relative health benefits arising from the changes in NO_2_ associated with these LULC modifications.

## 2. Materials and Methods 

**Figure 1:**
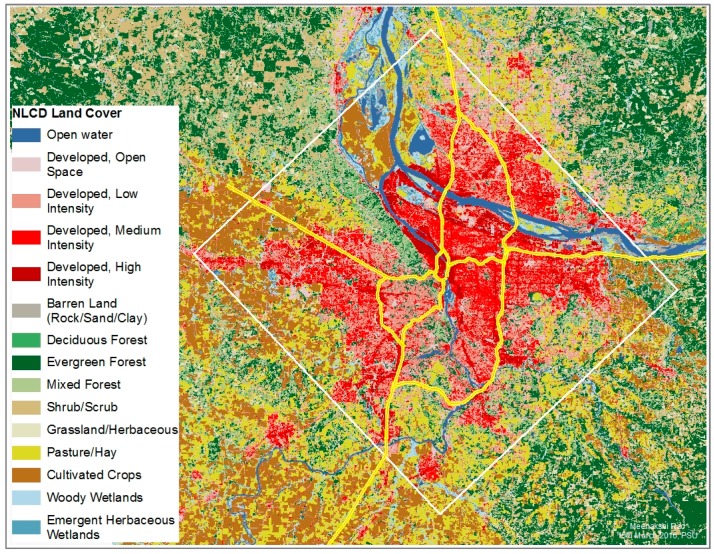
Land use in the Portland-Hillsboro-Vancouver area (based on NLCD 2011).

Our study area is the Portland-Hillsboro-Vancouver urban area, a mid-size metropolitan area located in the US Pacific northwest. Just over 1.8 million people reside within the study area, which encompasses 2350 km^2^. This area has diverse terrain—two rivers, mountains, and, like other urban areas, a wide mix of current land use. Within the study area, 6% of land area is high density development, 7.5% is developed open space, and 13% is forested area, based on the 2011 National Land Cover Database (NLCD) [63] and its land use categories (Figure 1, Table S1). According to the National Emissions Inventory (NEI) 2011 data, there are only three facilities in the study area permitted to emit >500 tons NO_x_ annually. Annual average daily traffic (AADT) for the freeways and state highways in the area ranges from 169,500 on I-84 to 610 on an inner city access ramp [64]. A brief overview of our methodology is provided below, with more details in the following subsections.

**Figure 2:**
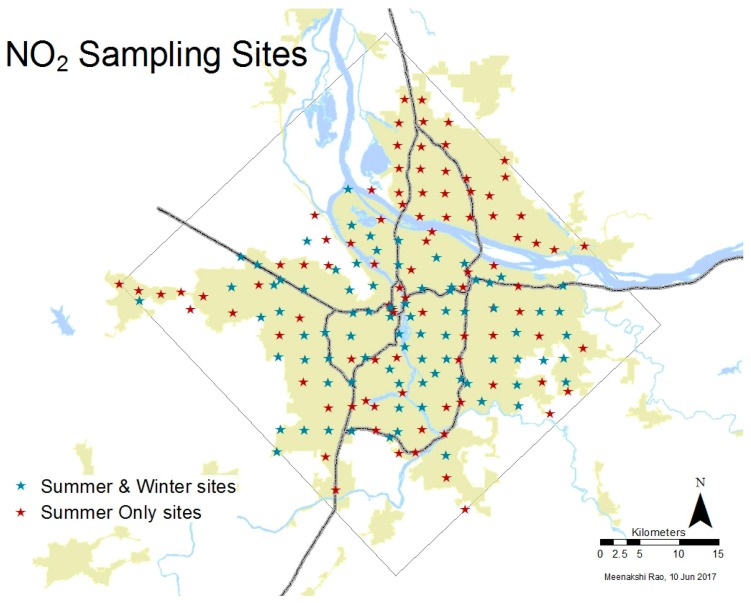
Summer and winter observation sites, with minimum bounding rectangle and urbanized area footprint. Blue stars represent sites monitored in summer and winter, red stars represent sites monitored in summer only.

NO_2_ was measured over two 2-week long field campaigns using relatively low cost passive chemical samplers made by Ogawa Co. (Pompano Beach, FL, USA). NO_2_ was sampled once during summer (22 August–6 September 2013) at 174 sites in the Portland-Hillsboro-Vancouver area. Due to the logistical and resource intensity of resampling, a randomly selected subset of 82 sites was sampled again in winter (13–27 February 2014) (Figure 2). Sites were chosen to capture the effect of roads, railroads and vegetation on ambient NO_2_.The average measured NO_2_ in summer was 11 ppb, with observed values ranging from 4 to 23 ppb, while the average measured winter NO_2_ was 13 ppb, with observed values ranging from 3 to 29 ppb. Additional details about the field campaign can be found in Rao et al. [26,65].

Datasets described in Table 1 were used to determine LULC variables. Care was taken to find datasets that were closest to the period of the field campaigns. Our analysis followed the steps below:

1. Conditional inference random forest was used to develop summer and winter NO_2_ models based on the NO_2_. These observationally-based summer and winter LURF models of NO_2_ were evaluated using both statistical measures as well as by comparison with LUR models, developed utilizing the same LULC variables.
2. A high spatial resolution, annual average NO_2_ model (required to estimate health impact using BenMAP) was developed by applying the summer and winter LURF models to a grid of points 200 m apart covering the study area, then averaging the summer and winter NO_2_ predictions at each grid point.
3. Next, the NO_2_ associated with each individual land use category was estimated by applying the summer and winter LURF models to modified LULC variables, with the summer and winter NO_2_ values being averaged to estimate annual average NO_2_ for the 200 m grid. Further, BenMAP [56], the health benefits assessment tool from the US EPA, was used to assess the respiratory impact of each LULC category. The health impact is assessed as the change in incidence in health outcomes arising from the change in NO_2_ between the NO_2_ in step 2 and in step 3.
4. Finally, after we had identified the LULC categories that have the greatest impact on ambient NO_2_ (from step 3) and identified ones that are amenable to change, a sensitivity analysis was undertaken in which these LULC categories were modified in the LURF model, and the change in NO*_2_* and the consequent respiratory health impact arising from each modification estimated.

These analyses are described in greater detail below. All spatial analyses were done in ESRI’s ArcMAP (version 10.2, ESRI, Redlands, CA, USA), health impact analyses in BenMAP (version 4.0.35 US EPA, Research Triangle Park, NC, USA), and the statistical analyses in the open source statistical program R version (version 3.1.1, R Foundation for Statistical Computing, Vienna, Austria) (10 July 2014) [61,62].

### 2.1. Developing the LURF Models

#### 2.1.1. Extracting Land Use Variables

LULC categories to be used as predictors in development of the LURF (and LUR) models were chosen either because they were known strong proxies for NO_2_ (e.g., freeways) or identified based on a literature review and our prior campaigns in the Portland area [26,33,34,35,66,67,68]. Table 1 lists the LULC data sets used, the data source, and the spatial resolution of the data. LULC variables were extracted in 12 buffers, ranging from 100 to 1200 m in radius (in 100 m increments) for each land use category, at each site (174 sites, as the winter sites were a subset of the summer sites). Latitude, longitude and elevation were also associated with each site. The NLCD categories deciduous, evergreen and mixed forest were added together to create a “trees” category. In all, ~200 land use variables were associated with each site. A randomly selected 25% of observations (42/174 for summer, 20/82 for winter) were set aside as a “validation” data set for model evaluation prior to the start of model development to enable a hold-out validation assessment for the LURF models. All model development was subsequently done on the remaining 75% “training” data set. 

#### 2.1.2. Developing the LURF Model

##### Random Forest

Random forest is an ensemble statistical learning method based on regression trees. Regression trees [44] divide the p-dimensional predictor space into p-dimensional rectangles, such that the total of the residual sum of squares over all the rectangles is minimized. The prediction for any set of predictors P_i_ is the average of all observations that fall in the rectangle containing P_i_. Regression trees tend to over-fit the training data, resulting in large variance, and hence potentially large prediction errors on unseen data. To address this issue, Brieman [45] developed the random forest methodology in which an ensemble of regression trees is created using bagging, that is by taking repeated samples with replacement from the training data set. Further, at each node for each tree in the forest, only a random subset of variables is considered for splitting, which results in decorrelated trees. Predictions are the average over all predictions for all trees in the forest for which the sample is out-of-bag. Strobl et al. [69] have further refined the methodology by using a conditional permutation scheme that corrects for the inflated variable importance of correlated predictors in random forest [70], which they call conditional inference random forest. All random forest model development for this study was done using conditional inference random forests as implemented in the “party” package version 1.0–23 [70,71,72] in R [73].

##### Developing the LURF and LUR Models

Summer and winter random forest NO_2_ models were developed using the ~200 LULC variables as predictors, in two phases. In the first phase, a conditional inference random forest, using the “party” package [70,71,72] in R [73], was used to identify the buffer size that was the most important predictor within each LULC category. Using only the most important buffer size for each land use category reduced the number of potential predictor variables from ~200 to ~20, and made the final model more interpretable. In the second phase, we again used conditional inference random forests, now with the reduced predictor set containing one buffer size for each LULC category, together with the point features latitude, longitude and elevation, to develop the observationally-based NO_2_ LURF models for summer and winter. Random forest models take a random seed and two hyper-parameters, namely number of regression trees to include in the random forest (ntree), and the number of variables to consider for a split at each node in each regression trees in the forest (mtry). We systematically explored the ntree (500, 1000, 2000, 3000, 4000, 5000) × mtry (1, 2, 3, 4, 5, 6, 7, 8) space for a range of random seeds to identify a robust LURF model for each season. 

The seasonal LUR models were developed used the same set of variables as the LURF seasonal models. We used a correlational matrix to narrow the variables to a smaller, less correlated subset. We next used stepwise regression, AIC, and VIF to identify four models, then used k-fold (k = 6) to identify the final LUR model for summer and winter.

These observationally-based summer and winter random forest models were each applied to points on a 200 m grid covering the study area. The 200 m resolution was chosen as a balance between computational time and intensity of extracting the LULC variables at each grid point, and the number of grid points required for higher spatial resolution. These seasonal fine-spatial scale NO_2_ models for the Portland-Hillsboro-Vancouver area were then averaged to develop the annual average NO_2_ LURF model.

### 2.2. Assessing the Performance of the LURF Model

Performance of the summer and winter LURF models was assessed using statistical performance measures for both model fit and predictive ability on unseen data. The statistical performance metrics used for model assessment are goodness of fit (R^2^); normalized mean bias (=1/N * Σ {[modeled (NO_2_) − observed(NO_2_)]/[observed(NO_2_)]}); and normalized mean error (=1/N * Σ {abs[modeled (NO_2_) − observed(NO_2_)]/[observed(NO_2_)]}). The normalized mean bias is an estimate of systematic over- or under-estimation of the LURF models as compared to the observations, while the normalized mean error is the estimate of the average difference in the NO_2_ predicted by the models and observations.

The predictive ability of the summer and winter LURF models was gauged by computing the root mean square error (RMSE) of the NO_2_ predicted for the validation data with respect to the observations. Since the validation data sets are not used in model development, the validation RMSEs provide a good estimation of model performance on unseen data. The LURF validation data set RMSEs were further compared with RMSEs reported in the literature for LUR models, as well as the validation RMSEs of the LUR models developed using the same training, validation, and land use data sets as the LURF model. 

### 2.3. Association of Current LULC, Ambient NO_2_, and Respiratory Health

We estimated the influence of each LULC category on ambient NO_2_ concentrations with a simple analysis: each land use category under consideration was set to zero over the entire study area, while keeping the remaining land use variables unchanged. Summer and winter NO_2_ predictions for the 200 m grid were averaged to estimate the annual impact of each land use category. The difference in modeled NO_2_ concentrations between the annual model and the model with the LULC category set to zero was used as an indicator of the NO_2_ associated with that LULC category. In essence, we simulated the annual average NO_2_ concentrations across the urban landscape under the assumption that each LULC category was replaced by an NO_2_-neutral land use; estimating, for example, the annual average NO_2_ concentrations if there were no trees in the Portland-Hillsboro-Vancouver area or if there were no traffic on the highways. Once we had estimated the NO_2_ associated with each land use category, we estimated the respiratory health impacts associated with this LULC-associated NO_2_ in terms of incidence rates and economic valuations using BenMAP. Table S2 lists the health impact functions and valuation methods from BenMAP that were used in this evaluation. Population within each 200 m grid cell in 2013 was estimated using Popgrid, an ancillary program to BenMAP, based on block-group level population from the 2000 US Census, projected to 2013. 

### 2.4. Evaluating the Mitigation Potential of LULC Modifications

Once we had determined the LULC categories with the strongest association with NO_2_, We undertook a sensitivity analysis to estimate the relative impact of modifications to these LULC categories on ambient NO_2_, and consequently, on respiratory health. We focused on four LULC categories, namely vehicle miles traveled on freeways, tree canopy, high intensity development, and open development. These categories were chosen as they were shown to be associated with NO_2_ and are amenable to planned change: many city climate action plans [23,24,25] already incorporate targets for VMT, tree canopy and impervious areas; and previous research has shown that they have a discernible impact on ambient NO_2_ concentrations [26,74]. For all these categories, we considered changes of ±2%, ±5% and ±10% to the LULC feature. For tree canopy, high intensity and open development, the percentage change is based on the buffer size, so that a 2% increase results in an increase even at grid points that currently didn’t have any of these land covers in their vicinity. In case of an increase, other LULC features in the vicinity of the point were proportionately decreased; while in the case of a decrease, the other LULC features were proportionately increased. However, care was taken that the modified land use did not go below 0% or above 100%. Health benefits (or dis-benefits), arising from the NO_2_ changes associated with each LULC modification in the sensitivity analysis were estimated using BenMAP.

## 3. Results

### 3.1. Assessing the Performance of the LURF Models

**Figure 3:**
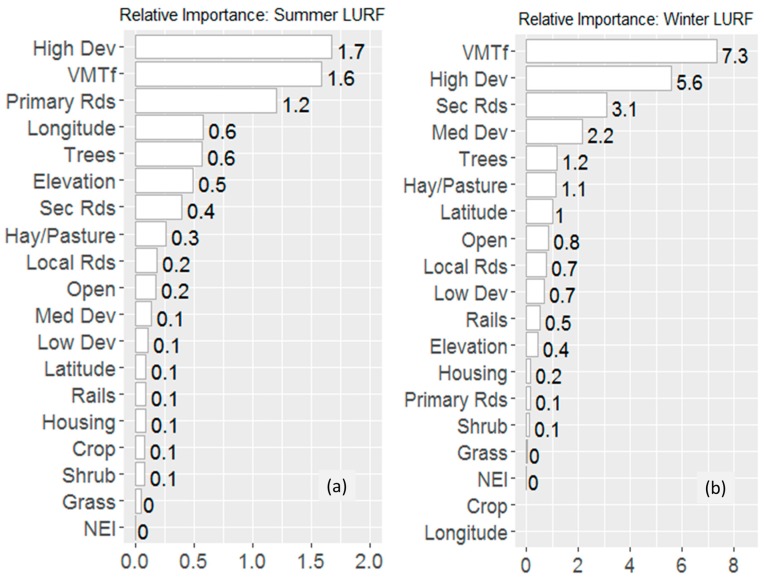
Relative importance of the LULC predictors (**a**) in the summer and (**b**) winter LURF models.

**Figure 4:**
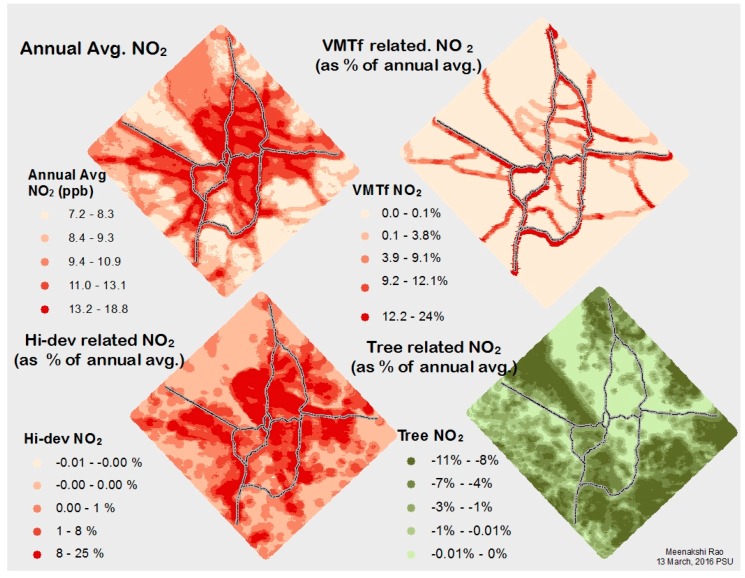
Percentage change in annual average NO_2_ when a land use/land cover category is replaced with a neutral land use.

The relative importance of the LULC predictors for the summer and winter LURF models can be found in Figure 3; and the LURF-derived 200 m-resolution map of annual average NO_2_ in the Portland-Hillsboro-Vancouver area can be seen in Figure 4. Based on statistical measures using hold out validation, the summer and winter random forest models perform well with an R^2^ of 0.80 and 0.83 respectively, indicating that a high degree of variance is captured by the models [7]. Both summer and winter LURF models show non-zero normalized mean bias and normalized mean error: the summer and winter LURF models show mean biases of 9% and 12% respectively; and mean errors of 20% and 24%. Thus, the LURF models systematically overestimate NO_2_ concentrations as compared to the observations (Table 2). These findings are consistent with 10-fold validation as well (Table S3).

The predictive ability of the LURF models, as gauged by the validation data RMSEs, indicates that the summer and winter models, on average, predict NO_2_ concentrations within 2.4 ppb of the measured NO_2_ in summer, and 3.8 ppb in winter (the higher winter validation RMSE being consistent with fewer winter observations). These RMSEs are consistent with RMSEs for NO_2_ LUR models in the literature, in fact lying towards the lower end of the reported range of 1.4–34 ppb [75]. The RMSEs for the summer and winter LURF models of 2.4 ppb and 3.8 ppb are also on par with the validation RMSEs of 2.8 and 3.4 for summer and winter LUR models developed using the same data sets (Table 2). However, it is important to note that both the LUR and LURF models overestimate the annual average concentration of NO_2_ at the DEQ monitoring station: 13.3 ppb and 13.4 ppb respectively, as compared to the annual average of 9.4 ppb based on the DEQ observations.

The annual average NO_2_ predicted by the LURF differs from the bias-corrected LURF model by 4% (Figure S1). We next compare the LURF and LUR models for the Portland-Hillsboro-Vancouver metropolitan area: these are is highly correlated, with a Pearson correlation coefficient of 0.85 (Figure S1). The relationship between LUR and LURF predicted NO_2_ can be summarized by the best fit regression line which has an adjusted R^2^ of 0.72 (Figure S1), indicating an effective linear mapping from LUR predicted NO_2_ to LURF predicted NO_2_ concentrations. The annual NO_2_ predictions using random forest show a systematic overestimation as compared to the LUR predictions, with a 13% mean bias. This bias can be explained by the difference between the regression and random forest methodologies. Regression determines the best-fit slopes or coefficients based on minimizing distance from a curve in space, while random forest (an ensemble of regression trees) works on similarity, assigning an outcome based on the average of “similar” observations. Thus, in a regression model, predicted values can lie outside the observed range of values, while in a random forest model, predicted values are restricted to lie within the observed range. This results in an overestimation by LURF, as compared to LUR, at the lower end, and an underestimation at the upper end of predictions, as can be clearly seen in Figure S1. The mean error is 22%; that is, on average there is a 22% difference between the NO_2_ concentrations predicted by the LURF and LUR models (Table 2), a difference which is on par with the mean error of both the LUR and LURF models with respect to observations.

### 3.2. Association of Current LULC, Ambient NO_2_, and Respiratory Health

Figure 4 shows the local variation in annual average NO_2_ in the Portland-Hillsboro-Vancouver area, as well as the local spatial variation of NO_2_ associated with three LULC categories: VMT*f*, high-intensity development, and trees. Modeled, annual average concentrations of NO_2_ within the study area range from 7 to 19 ppb, well below the US EPA standard (53 ppb) [76], though near the World Health Organization standard (20 ppb) [77].

Of the 11 land use categories considered, eight (high intensity development, VMT*f*, primary, secondary & local roads, railroads, housing density, and permitted NO_2_ emissions) contribute to increasing ambient NO_2_. The remaining three land use categories (developed open spaces, trees, shrubs) are associated with decreasing ambient NO_2_ concentrations (Table 3). The changes in ambient NO_2_ range from a decrease of 0.4 ppb associated with trees to an increase of 0.9 ppb associated with roadways, when averaged over the study area.

Estimated annual incidence of respiratory health problems linked to LULC-related NO_2_ concentrations are shown in Table 4, while the annual economic valuation of these health impacts can be found in Table S4. The health burden of NO_2_, based on the health impact functions in the BenMAP database, appears to fall disproportionately on children under 14 years old, primarily in the form of asthma exacerbation and missed school days. For instance, there are 42,000 incidents of asthma exacerbation for every 100,000 4–12 year-olds, of which approximately 8% may be associated with VMT*f*, and −3% with tree canopy.

### 3.3. Evaluating the Mitigation Potential of LULC Modifications

**Figure 5:**
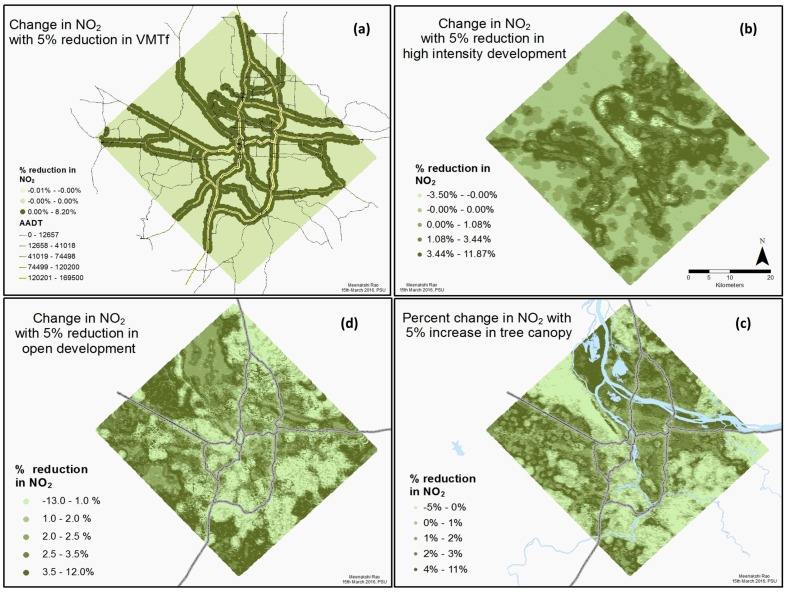
The spatial distribution and magnitude of the change in modeled NO_2_ concentrations in response to a ±5% change in (clockwise from top left) (**a**) VMT*f* (**b**) high intentsity development (**c**) tree canopy (**d**) open development.

Figure 5 and Table S5 present the results of the sensitivity analysis, showing how local NO_2_ concentrations change in response to modifications in four LULC categories: VMT*f*, high intensity development, open development and tree canopy. Table S5 summarizes the percent change in local annual NO_2_ (averaged over the entire study area) in response to ±2%, ±5% and ±10% changes in these LULC categories. We see that changes in VMT*f* have relatively little impact on the region-wide average of NO_2_, while increasing tree cover by 10% decreases local concentrations of NO_2_ by 3%, on average, across the study area. The reason for the relatively small change in NO_2_ in response to an increase in VMT*f* (0.1%) versus the comparatively larger changes in regionally averaged NO_2_ in response to increases in high intensity development (2–11%), trees (1–3%), and open development (1–3%) (Table S5) is partly methodological: high intensity, tree, and open areas are increased as a percentage of the buffer area, and not as a percentage of the LULC category within the buffer. For example, a grid point with 0% tree cover, tree canopy is increased to 2%, 5% and 10% in the sensitivity analysis, similarly for a point with 20% tree cover, the increases are to 22%, 25% and 30%. This method of changing LULC categories results in effects observed across the entire study area.

Figure 5 shows the spatial pattern and relative magnitude of NO_2_ change associated with a 5% change in each of the four LULC categories. We see that the spatial pattern of NO_2_ response to LULC modifications is distinct for the four LULC categories considered. The NO_2_ change in response to changes in VMT*f* is constrained to a narrow buffer around the freeways, consistent with the observed drop-off in NO_2_ concentrations with distance from roads [3]. The response of NO_2_ to modifications in the other three categories is much more spread out across the study area. For high intensity development, the greatest percentage reduction in NO_2_ is towards the center of the study area, where the high density development is most intense, possibly reflecting the association of combustion processes and development. For developed open space, on the other hand, there is an increase in NO_2_ on the periphery of the study area, consistent with treed areas on the outskirts being replaced with open development; and a decrease in NO_2_ towards the center, due to high intensity development being replaced by open development. Similarly, we observe a small increase to no change in NO_2_ concentrations in changing tree canopy in areas which already have high percentage of tree cover (see Figure 1), and a decrease in NO_2_ in areas with previously low tree canopy. Local impact of a 5% change in VMT*f*, high intensity development, open development and tree canopy on NO_2_ is as high as 8%, 12%, 12% and 11% respectively. Together these indicate that different LULC modification strategies might be optimum in different parts of the urban area for mitigating local NO_2_.

With this modeled estimate of the change in NO_2_ in response to LULC modifications, we next estimated the health benefit associated with this NO_2_ change using BenMAP. We focused on change in incidence rates of asthma exacerbation in 4–12 year olds arising from the change in local NO_2_ corresponding to changes in VMT*f* and tree canopy. Table 5 shows the percent change in annaul incidence rates of asthma exacerbation (as compared to the incidence rate for all NO_2_-related asthma exacerbation of ~42,000, Table 3), arising from the change in local NO_2_ associated with modifications to VMT*f* and tree canopy. Results shown are averaged over the study area, as well as averaged over just the area lying within the worst NO_2_ quintile (Figure S2). We see that decreasing VMT*f* even by 10% has very little impact on asthma incidence rates, while increasing tree cover is associated with up to an 11% decrease in the incidence of all NO_2_-related asthma (total NO_2_-related asthma being about ~42,000 incidents/100,000, Table 4). Although this result may initially seem surprising, it is a consequence of the fact that health outcomes depend on the distribution of the air pollutant as well as the population. Although decreasing VMT*f* by 10% decreases NO_2_ substantially in a 700 m around the freeways, the population of 4–12 year-olds in this narrow buffer is small, leading to a small change incidence rates.

## 4. Discussion

We showed that the random forest ensemble learning technique performed well in capturing the fine spatial scale variation of NO_2_—the LURF model performed well based on statistical performance metrics and predictive ability, on par with the widely used LUR methodology. Although the LURF model overestimates NO_2_ concentrations (Table 2), the ability of LURF to explore impacts of individual LULC variables suggests that the random forest technique can be added to the repertoire of new and established statistical such as structural equation modeling [78], generalized boosting models [19], and neural networks [18,79] that are used today to better understand air pollution in our urban environments; and particularly to explore how cities can modify land use to reduce NO_2_ and improve respiratory health. We should remain cognizant of the fact that the LURF models, like the LUR models, are likely not to transfer well between cities, especially cities in different parts of the world [80,81].

We found—based on the BenMAP health impact functions—that even in the Portland-Hillsboro-Vancouver metropolitan area, a city in compliance with US EPA and WHO standards, there still exists a significant respiratory burden, borne predominantly by children under the age of 12, resulting from the ubiquitous urban pollutant NO_2_. Utilizing the LURF NO_2_ model, we were able to examine the relative impact and spatial pattern of the different urban LULC categories on incidence rates of respiratory health issues. For example, the LURF model showed that NO_2_ associated with VMT*f* was linked to an increase in respiratory health issues (~3200 per 100,000 increase in asthma exacerbation symptoms in 4–12 year olds), and further, this impact was spatially clustered close to freeways and highways. The overall effect of urban tree canopy was smaller (~1369 per 100,000 decrease in asthma exacerbation in 4–12 year olds) and but more widely spatially distributed over the study area.

Today, municipalities often engage in city-wide LULC modifications such as VMT reduction and “greening” campaigns, both to reduce emissions and improve human health. However, no clear and accessible assessment methodology has existed to help estimate the benefits accruing from these campaigns, or alternately, to identify optimum strategies from a range of strategies. Our sensitivity analysis using the LURF model to study the response of ambient NO_2_ to LULC modifications showed that planting trees locally (which has the effect of reducing developed areas and increasing tree canopy) may be a good strategy to reduce local NO_2_ concentrations and improve respiratory health. Furthermore, model outcomes suggest that a 10% increase in canopy coverage city-wide may reduce the incidences of childhood asthma by an order of magnitude more than a 5% or even 10% reduction in VMT*f*.

To the extent that the Portland-Hillsboro-Vancouver metropolitan area is representative of other mid-size cities in the USA, we can expect a similar burden of respiratory health due to NO_2_, borne disproportionately by children, in other cities as well [82,83,84,85]. Recent studies show that childhood exposure to traffic-related air pollution may lead to impaired lung function in early adulthood [82,86] and that exposure to air pollution in childhood is linked to poorer performance in school [87] which in turn could lead to lower earnings potential in adulthood. Given the increasing number of studies that suggest the role of the urban forest in promoting physical and mental well-being [88,89,90,91,92], it seems likely that small-scale strategic tree planting campaigns in either high NO_2_ areas and/or near roadways, or city-wide greening campaigns, may well play an important role in improving human health, which may come in some small part due to the mitigating of respiratory distress associated with NO_2_.

Keeping in mind that correlation does not imply causation, it is prudent that we seek to better understand the mechanisms through which land use modifications, including tree plantings, affect ambient NO_2_ concentrations. For example, trees have been shown to remove NO_2_ from the atmosphere through dry deposition [93,94]; yet the rates of dry deposition in the urban environment [28,95,96], species-specific dry deposition rates [97], seasonal variations in dry deposition, and other questions have yet to be studied extensively. We hope that this paper inspires further research, both statistical and mechanistic, into how urban land use design and modifications can be used to mitigate the health effects of urban air pollution.

## 5. Conclusions

In today’s rapidly urbanizing world, where, according to the World Health Organization, air pollution has become “the single largest environmental health risk” [1] there is an urgent need to design cities that promote cleaner atmospheres. Critically, the random forest technique applied in this paper is robust in handling noisy and missing data, a not uncommon feature of dense sensor networks, making it ideally suited for analyzing the flood of data from sensor technologies that are currently on the horizon. Since it is relatively easy to use, does not require intense computational support, and the output models are readily interpreted, the use of this technique has the potential to include a wide range of stakeholders, including planners, citizens, and agencies, in the process of better characterizing and managing local LULC to optimize air quality. 

The results presented here serve to highlight the need for future research to better understand the mechanisms that determine how different LULC categories shape the intra-urban patterns of air pollution within our cities. Combining the sophistication of new sensor technologies with advanced modeling techniques, including random forest, will contribute to a better understanding of the linkages between land use and urban air pollution and lead to creating healthier cities and more sustainable urban atmospheres for all.

## Figures and Tables

**Table 1:** Land use/land cover categories used in analysis, data source, and spatial resolution.

Land Use/Land Cover	Data Source
Housing	US Census Bureau, 2010 (block level)
Land cover classes (developed open space, high intensity development, trees, shrub/scrub, grassland, pasture, cultivated crops)	National Land Cover Database (NLCD), USGS, 2011 (30 m)
Permitted NO_2_ emissions	National Emissions Inventory, EPA, 2011 (point sources)
Elevation	USGS, 1/3 arc-second
AADT	NHPN (2010)
Roads (primary, secondary and local)	US Census Bureau, Tiger/Line (2013)
Latitude & Longitude	Google Earth, ArcMAP

**Table 2:** Performance metrics for the summer and winter LURF and LUR models using hold out validation.

Season and Model	Goodness of Fit	Model Bias		Prediction Error	
	Adj R^2^	Normalized Mean Bias	Normalized Mean Error	Validation MAE (NO_2_ ppb)	Validation RMSE (NO_2_ ppb)
**Summer**					
LUR	0.75	5%	20%	2.3	2.8
LURF	0.80	9%	20%	2.0	2.4
**Winter**					
LUR	0.80	5%	18%	2.5	3.4
LURF	0.83	12%	24%	2.8	3.8

**Table 3:** Estimated association of land use and annual average NO_2_ concentrations, averaged over the study area, as well as average land use values within the model buffers.

LULC Category	NO_2_ (ppb) Associated with Land Use	Range NO_2_ (ppb)	Typical LULC Values within Model Buffer	Range LULC Values within Model Buffer
Development, high-density	0.7	0–3.8	0.76 km^2^	0–7.9 km^2^
Roadways	0.9	0–6.2		
Vehicle Miles travelled on highways	0.4	0–3.5	133,916	0–1,329,013
Primary Roads	0.1	0–0.9	1.7 km	0–20 km
Secondary Roads	0.2	0–1.9	4.6 km	0–44 km
Local Roads	0.2	0–0.81	70 km	1.5–242 km
Railroads	0.1	0–0.6	4.3 km	0–60 km
Housing	0.1	0–0.7	42,917	5–311,582
Permitted NO_2_ emissions	0.0	0–0.0	19 tons/year	0–1064 tons/year
Developed open space	−0.3	−0.9–0	0.24 ha	0–3 ha
Trees	−0.4	−1.0–0	6.7 ha	0–50 ha
Shrub/Scrub	−0.1	−0.2–0	24 ha	0–495 ha

**Table 4:** Estimated annual incidence of respiratory problems per 100,000 individuals associated with LULC due to local influence on ambient NO_2_, in the Portland-Hillsboro-Vancouver urban area.

Health Impact	Annual Incidence Rate (per 100,000) Associated with LULC Category						
	All NO_2_	VMT*f*	Sec. Rds	High Intensity Dev.	Med. Intensity Dev.	Open Dev.	Trees
Asthma Exacerbation, Missed school days (4–12 year olds)	14,455	1109	1322	2393	1587	−583	−472
Asthma Exacerbation, One or More Symptoms (4–12 year olds)	42,171	3220	3837	6950	4606	−1692	−1369
Cough (7–14 year olds)	12,070	926	1108	1989	1338	−503	−414
Emergency Room Visits, Asthma (75 years and older)	22	2	2	3	2	−1	−1
Hospital admissions, Asthma (younger than 30 years)	1	0	0	0	0	0	0
Hospital admissions, Asthma (30 years and older)	1	0	0	0	0	0	0
Hospital admissions, Chronic Lung Disease (less Asthma) (65 years and older)	64	6	6	11	6	−2	−2
Hospital admissions, All Respiratory (65 years and older)	137	12	13	23	13	−5	−4

**Table 5:** Estimated change in the incidence of NO_2_-related asthma exacerbation associated with modifications to the two LULC categories VMTf and trees.

% Change in NO_2_-Related Asthma Exacerbation Symptoms in 4–12 Year Olds Due to Changes in NO_2_ Associated with LULC Modifications				
LULC Category/LULC Change	VMT*f*	VMT*f* (in Worst NO_2_ Quintile)	Trees	Trees (in Worst NO_2_ Quintile)
10% decrease	−0.5%	−0.8%	2%	1%
5% decrease	−0.2%	−0.4%	2%	1%
2% decrease	−0.1%	−0.2%	1%	1%
2% increase	0.1%	0.1%	−3%	−3%
5% increase	0.2%	0.3%	−6%	−6%
10% increase	0.4%	0.7%	−10%	−11%

## Supplementary Materials

The following are available online at www.mdpi.com/1660-4601/14/7/750/s1, Table S1: NLCD land use/land cover categories; Table S2: BenMAP health impact and valuation functions used in this study; Table S3: Performance metrics for the summer and winter LURF and LUR models using 10-fold validation; Table S4: Economic valuation of LULC-related NO_2_ health impacts; Table S5: Modeled mean and median of the NO_2_ (over study area) from a sensitivity analysis; Figure S1: % difference in NO_2_ as estimated using the biased and bias-corrected LURF models; Figure S2: LURF NO_2_-LUR NO_2_ plot and best fit line.
