# Supplementary material for: Assessing the Potential of Land Use Modification to Mitigate Ambient NO2 and Its Consequences for Respiratory Health
Source: Int J Environ Res Public Health. 2017 Jul 10;14(7):750. doi: 10.3390/ijerph14070750 (PMC5551188; doi:10.3390/ijerph14070750)
Supplement: Supplementary file 1 [file ijerph-14-00750-s001.pdf]

International Journal of
Environmental Research and Public Health

MDPI

Supplementary Materials

Assessing the Potential of Land Use Modification to Mitigate Ambient NO_2 and Its Consequences for Respiratory Health

Meenakshi Rao, Linda A. George, Vivek Shandas and Todd N. Rosenstiel

Table S1. NLCD land use/land cover categories.

Code	LULC	Description
11	Open water	All areas of open water, generally with less than 25% cover or vegetation or soil
12	Perennial Ice/Snow	All areas characterized by a perennial cover of ice and/or snow, generally greater than 25% of total cover.
21	Developed, Open Space	Includes areas with a mixture of some constructed materials, but mostly vegetation in the form of lawn grasses. Impervious surfaces account for less than 20 percent of total cover. These areas most commonly include large-lot single-family housing units, parks, golf courses, and vegetation planted in developed settings for recreation, erosion control, or aesthetic purposes.
22	Developed, Low Intensity	Includes areas with a mixture of constructed materials and vegetation. Impervious surfaces account for 20-49 percent of total cover. These areas most commonly include single-family housing units.
23	Developed, Medium Intensity	Includes areas with a mixture of constructed materials and vegetation. Impervious surfaces account for 50-79 percent of the total cover. These areas most commonly include single-family housing units.
24	Developed, High Intensity	Includes highly developed areas where people reside or work in high numbers. Examples include apartment complexes, row houses and commercial/industrial. Impervious surfaces account for 80 to 100 percent of the total cover.
31	Barren Land (Rocks/Sand/Clay)	Barren areas of bedrock, desert pavement, scarps, talus, slides, volcanic material, glacial debris, sand dunes, strip mines, gravel pits and other accumulations of earthen material. Generally, vegetation accounts for less than 15% of total cover.
41	Deciduous Forest	Areas dominated by trees generally greater than 5 meters tall, and greater than 20% of total vegetation cover. More than 75 percent of the tree species shed foliage simultaneously in response to seasonal change.
42	Evergreen Forest	Areas dominated by trees generally greater than 5 meters tall, and greater than 20% of total vegetation cover. More than 75 percent of the tree species maintain their leaves all year. Canopy is never without green foliage.
43	Mixed Forest	Areas dominated by trees generally greater than 5 meters tall, and greater than 20% of total vegetation cover. Neither deciduous nor evergreen species are greater than 75 percent of total tree cover.
51	Dwarf Scrub	Alaska only areas dominated by shrubs less than 20 centimeters tall with shrub canopy typically greater than 20% of total vegetation. This type is often co-associated with grasses, sedges, herbs, and non-vascular vegetation.
52	Shrub/Scrub	Areas dominated by shrubs; less than 5 meters tall with shrub canopy typically greater than 20% of total vegetation. This class includes true shrubs, young trees in an early successional stage or trees stunted from environmental conditions.

71	Grassland/Herbaceous	Areas dominated by grammanoid or herbaceous vegetation, generally greater than 80% of total vegetation. These areas are not subject to intensive management such as tilling, but can be utilized for grazing.
72	Sedge/Herbaceous	Alaska only areas dominated by sedges and forbs, generally greater than 80% of total vegetation. This type can occur with significant other grasses or other grass like plants, and includes sedge tundra, and sedge tussock tundra.
73	Lichens	Alaska only areas dominated by fruticose or foliose lichens generally greater than 80% of total vegetation.
74	Moss	Alaska only areas dominated by mosses, generally greater than 80% of total vegetation.
81	Pasture/hay	Areas of grasses, legumes, or grass-legume mixtures planted for livestock grazing or the production of seed or hay crops, typically on a perennial cycle. Pasture/hay vegetation accounts for greater than 20 percent of total vegetation.
82	Cultivated Crops	Areas used for the production of annual crops, such as corn, soybeans, vegetables, tobacco, and cotton, and also perennial woody crops such as orchards and vineyards. Crop vegetation accounts for greater than 20 percent of total vegetation. This class also includes all land being actively tilled.
90	Woody Wetlands	Areas where forest or shrub land vegetation accounts for greater than 20 percent of vegetative cover and the soil or substrate is periodically saturated with or covered with water.
95	Emergent Herbaceous Wetlands	Areas where perennial herbaceous vegetation accounts for greater than 80 percent of vegetative cover and the soil or substrate is periodically saturated with or covered with water.

Table S2. BenMAP health impact and valuation functions used for assessing incidence and economic value of NO_2 exposure in the Portland-Vancouver urbanized area.

Health Impact	Study	Valuation Method
Asthma Exacerbation, Missed school days (4 to 12 years)	O'Connor, George T., et al. "Acute respiratory health effects of air pollution on children with asthma in US inner cities." *Journal of Allergy and Clinical Immunology* 121.5 (2008): 1133–1139.	WTP: 1 symptom-day, Dickie and Ulery (2002) ǀ 0–17
Asthma Exacerbation, One or More Symptoms (4 to 12 years)	O'Connor, George T., et al. "Acute respiratory health effects of air pollution on children with asthma in US inner cities." *Journal of Allergy and Clinical Immunology* 121.5 (2008): 1133–1139.	WTP: 1 symptom-day, Dickie and Ulery (2002) ǀ 0–17
Cough (7 to 14 years)	Schwartz, Joel, et al. "Acute effects of summer air pollution on respiratory symptom reporting in children." *American journal of respiratory and critical care medicine* 150.5 (1994): 1234–1242.	WTP: 3 symptoms 1 day, Dickie and Ulery (2002). ǀ 18–99
Emergency Room Visits, Asthma (all ages)	Ito, Kazuhiko, George D. Thurston, and Robert A. Silverman. "Characterization of PM2. 5, gaseous pollutants, and meteorological interactions in the context of time-series health effects models." *Journal of Exposure Science and Environmental Epidemiology* 17 (2007): S45–S60.	COI: Standford et al. (1999) ǀ 0–99
HA, All Respiratory (65 years and older)	Yang, Qiuying, et al. "Association between ozone and respiratory admissions among children and the elderly in Vancouver, Canada." *Inhalation toxicology* 15.13 (2003): 1297–1308.	
HA, Asthma (29 years or younger)	Linn, William S., et al. "Air pollution and daily hospital admissions in metropolitan Los Angeles." *Environmental health perspectives* 108.5 (2000): 427.	COI: med costs + wage loss ǀ 0–99

HA, Asthma (30 years or older)	Linn, William S., et al. "Air pollution and daily hospital admissions in metropolitan Los Angeles." *Environmental health perspectives* 108.5 (2000): 427.	COI: med costs + wage loss ∣ 0–99
HA, Chronic Lung Disease (less Asthma) (65 years and older)	Yang, Qiuying, et al. "Effect of short-term exposure to low levels of gaseous pollutants on chronic obstructive pulmonary disease hospitalizations." *Environmental Research* 99.1 (2005): 99–105.	

Table S3. Performance metrics for the summer and winter LURF and LUR models using 10-fold validation.

	**Goodness of fit**	**Model bias**		**Prediction error**	
	Adj R^2	Normalized Mean bias	NormalizedAbsolute mean error	Validation MAE (NO_2 ppb)	Validation RMSE (NO_2 ppb)
**Summer**					
LUR	0.72	6%	20%	2.0	2.4
LURF	0.80	7%	19%	2.2	2.7
**Winter**					
LUR	0.77	5%	19%	2.4	3.0
LURF	0.83	11%	21%	3.1	4.0

Table S4. Estimated economic valuation of respiratory health impact associated with LULC due to local influence on ambient NO_2 concentrations, for the Portland-Vancouver urban area.

**Health Impact**	**Valuation in $1,000s 2013 USD**						
	All NO_2	VMT*f*	Sec roads	High intensity dev	Med intensity dev	Open dev	Trees
Asthma Exacerbation, Missed school days (4–12 year olds)	$7,290	$559	$667	$1,207	$800	−$294	−$238
Asthma Exacerbation, One or More Symptoms (4–12 year olds)	$21,266	$1,624	$1,935	$3,505	$2,323	−$853	−$691
Cough (7–14 year olds)	$3,219	$247	$296	$531	$357	−$134	−$110
Emergency Room Visits, Asthma (75 years and older)	$7	$1	$1	$1	$1	$0	$0
HA, Asthma (younger than 30 years)	$65	$5	$6	$11	$7	−$2	−$2
HA, Asthma (30 years and older)	$77	$6	$7	$13	$7	−$3	−$2
HA, Chronic Lung Disease (less Asthma) (65 years and older)	$2,633	$235	$264	$455	$268	−$101	−$90
HA, All Respiratory (65 years and older)	$7,752	$667	$758	$1,280	$748	−$281	−$247
Total:	$39,599	$3,345	$3,932	$7,001	$4,511	−$1,669	−$1,380

Table S5. The percent change in local NO_2, averaged over the Portland-Vancouver area, in response to land cover modifications.

**LULC change**	**VMT*f* average % change in NO_2**	**HDEV average % change in NO_2**	**TREE average % change in NO_2**	**OPEN average % change in NO_2**
10% decrease	−0.2%	−2.7%	1.4%	1.8%
5% decrease	−0.1%	−1.8%	0.9%	1.1%

2% decrease	−0.0%	−0.9%	0.4%	0.5%
2% increase	0.0%	1.7%	−1.0%	−1.1%
5% increase	0.1%	5.9%	−1.9%	−2.1%
10% increase	0.1%	10.9%	−3.1%	−3.3%

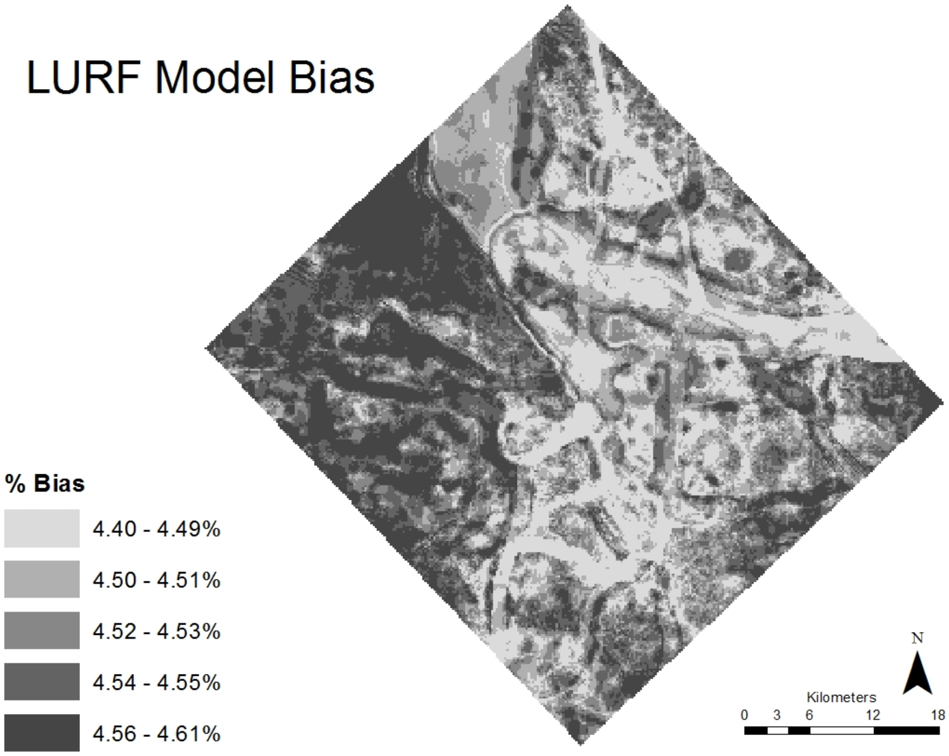

Figure S1. % difference in NO_2 as estimated using the biased and bias-corrected LURF models.

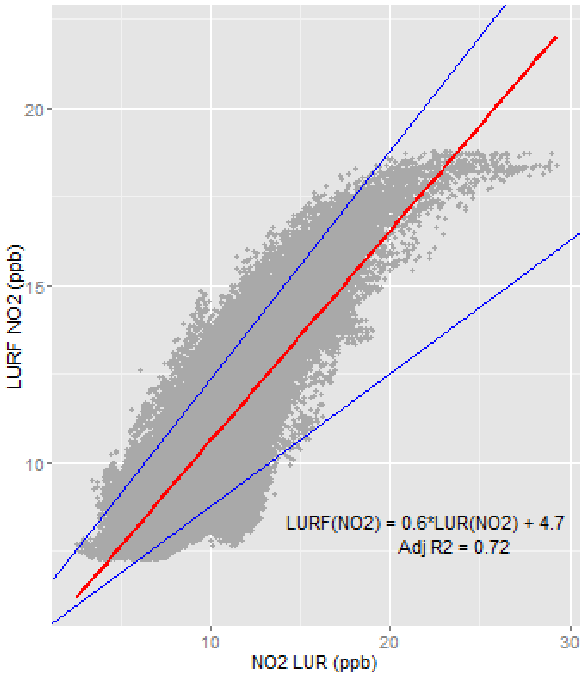

Figure S2. LURF NO_2-LUR NO_2 plot and best fit line (in red). The blue lines are the quantile regression lines for the 0.1 (below the red line) and 0.9 deciles (above the red line).
